# The Life Mission Theory III. Theory of Talent

**DOI:** 10.1100/tsw.2003.115

**Published:** 2003-12-11

**Authors:** Soren Ventegodt, Niels Jorgen Andersen, Joav Merrick

**Affiliations:** The Quality of Life Research Center, Copenhagen K, Denmark

**Keywords:** quality of life, QOL, philosophy, human development, holistic medicine, public health, Denmark

## Abstract

When we acknowledge our purpose as the essence of our self, when we take all our power into use in an effortless way, and when we fully accept our own nature — including sex and sexuality, our purpose of life takes the form of a unique talent. Using this talent gives the experience of happiness. A person in his natural state of being uses his core talent in a conscious, joyful, and effortless way, contributing to the world the best he or she has to offer. Full expression of self happens when a person, in full acceptance of body and life, with whole-hearted intension, uses all his personal powers to realize his core talent and all associated talents, to contribute to his beloved and to the world. Thus, self-actualisation is a result of a person fully expressing and realizing his core talent.The theory of talent states that a core talent can be expressed optimally when a human being takes possession of a three-dimensional space with the axis of purpose, power and gender, as we have a threefold need: 1-Acknowledging our core talent (our purpose of life) and intending it 2-Understanding our potential powers and manifesting them 3-Accepting our human form including our sex and expressing itThe first dimension is spiritual, the next dimension is mental, emotional and physical, and the third dimension is bodily and sexual. We manifest our talents in a giving movement from the bottom of our soul trough our biological nature onto the subject and object of the outer world. These three dimensions can be drawn as three axes, one saggital axis called purpose or love or me-you, one vertical axis called power or consciousness (light) or heaven-earth, and one horizontal axis called gender or joy or male-female. The three core dimensions of human existence are considered of equal importance for expression of our life purpose, life mission, or core talent. Each of the dimensions is connected to special needs. When these needs are not fulfilled, we suffer and if this suffering becomes unbearable we deny the dimension or a part of is. This is why the dimensions of purpose, power and gender become suppressed from our consciousness.

## INTRODUCTION

Everybody has, among a wide range of talents, a special gift, something this person can do or be better than anybody else in the world[1]. Everybody is special, even divine, in his core talent. This talent arises from the bottom of our human existence, as the essence of our human nature, the essence of our true self. When necessary, our core talent draws on our secondary talents, which again draw on tertiary talents and so forth, making all the talents in the world available for us, when they serve our true purpose of life[2].

Behind any talent is the intent of realizing this talent, but biology and the outer world have barriers that we need to overcome. This sometimes makes the process of developing your talent painful. The closer a talent is to the existential core, the stronger is the intent, and the stronger is the pain. This existential and emotional pain often brings the person to deny his core talent[1]. But the talent is still there, hidden below the surface of your existence. When we deny our core talent early in life, our whole personality is recentered on a secondary or even tertiary talent, thus giving rise to a false self, the ego[2]. From the position of a false self we cannot be as talented as we were meant to be.

## EXPRESSION OF TALENT

For us to flourish with our central talent and to obtain the state that Abraham Maslow[3] called self-actualization, we have a threefold need:

1. Acknowledging our core talent (our purpose of life) and intending it
2. Understanding our potential powers and manifesting them
3. Accepting our human form including our sex and expressing it

The first dimension is spiritual; the next dimension is mental, emotional, and physical; while the third dimension is bodily and sexual. We manifest our talents in a giving movement from the bottom of our soul through our biological nature onto the subject and object of the outer world.

These three dimensions can be drawn (Fig. 1) as three axes: one saggital axis called purpose or love or I-you, one vertical axis called power or consciousness (light) or heaven-earth, and one horizontal axis called gender or joy or male-female.

## THE THREE CORE DIMENSIONS OF HUMAN EXISTENCE

The three core dimensions of human existence are considered of equal importance for the expression of our life purpose, life mission, or core talent. Each of the dimensions is connected to special needs. When these needs are not fulfilled, we suffer and if this suffering becomes unbearable, we deny the dimension or a part of it. This is why the dimensions of purpose, power, and gender become suppressed from our consciousness

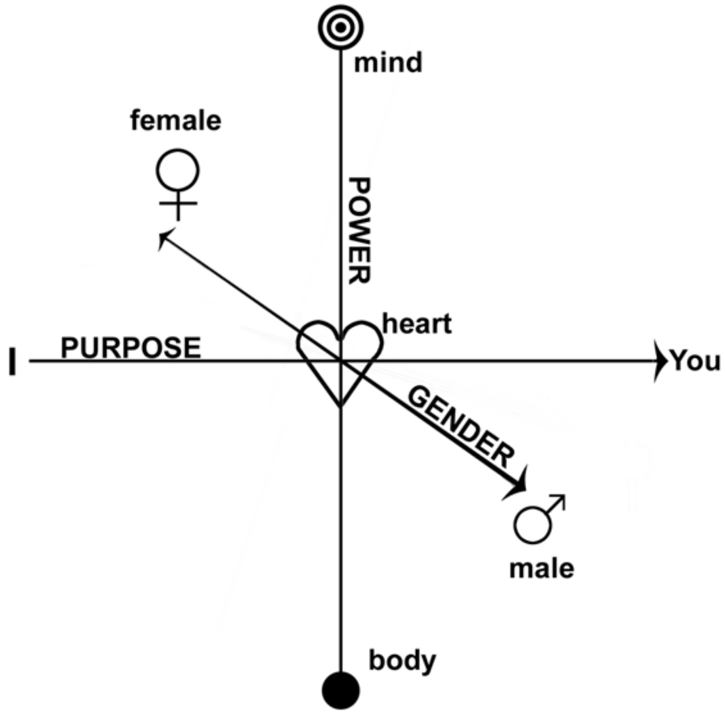

**FIGURE 1**. A human being exists in three dimensions, and all of them must be in their natural state to allow the full expression of talent. The saggital axis is called *purpose* or *love* or *I-you*, the vertical axis is called *power* or *consciousness* (light) or *heaven-earth*, and the horizontal axis is called *gender* or *joy* or *male-female*.

## Purpose

The saggital axis is called purpose or love or I-you. It carries our life purpose or mission of life[1]. Everybody is born with a great gift, something that we are meant to contribute to this world. When we use this gift, and give our very best to other people and to the world, we become truly happy. The central talent is the core in our healthy personality or the backbone of our sound existence. It draws on all the other talents in the vast human heritage. It arises from our wholeness (soul) and is so abstract and all-embracing that it comes in useful in all possible situations in life. The explanation for this is that our purpose of life is what structures our understanding, our perception, and our interpretation of reality. The purpose of life is what gives our life meaning. Our purpose, and the intent behind it, is directed forward against you and the intent, as expressed by Martin Buber (1878–1965)[4]. Only the person who is fully aware of his or her purpose of life can fully express his or her true talent.

To be able to see and acknowledge our purpose of life, we must be seen and acknowledged by others, first of all by our mother and father, later by other people. The need affiliated with the I-you axis is *acknowledgment of me*. I can support you on this axis by *acknowledging you*. I can help myself in this dimension by acknowledging myself.

## Power

To give is an action of power and in our natural state of being we are immensely powerful[3,4,5,6]. To bring your talent to the world takes a lot of power. There is a reason why the world is in need of what you have to give, so you cannot expect the world to accept your gift without a struggle. To impact other people or to make a change in the world, you must fight, and for this fight you need power. So without power, you cannot be truly talented and you cannot make any difference or any important contribution to the world. To influence another subject, you must use a lot of different talents bound to mind, feelings, and body.

All parts of the body must be in fine shape, the organs and cells of the body must be in their natural state. All the qualities connected to mind and brain, to feelings and emotions, and the physical body and its life must be drawn on. We are talking about qualities like value, joy, respect, love, courage, expression of self, attention, intelligence, patience, awareness, and maybe the most fundamental talent called coherence[6]. Coherence makes us an integral part of the world, mankind, and the global ecosystem. Only the person who is fully alive on the vertical axis of power and consciousness can fully express his or her talent. The concept of a “spiritual warrior” describes this accurately.

To realize and develop our powers, we need awareness of our mind, respect for our feelings, and care for our body. The needs affiliated with the power-axis are *awareness*, *respect*, and *care.* I can support you on this axis if I am *aware, respectful,* and *caring*[7]. I can develop in this dimension by being aware, respectful, and caring and by thorough self-examination.

### Gender

Surprisingly, a person cannot express his or her true talent without a total acceptance of his or her own gender[8]. The reason for this is twofold: the dimension of gender is also the dimension of joy, so it seems that the power of the body cannot manifest itself without an accurate and well-defined male or female appearance. The simple explanation is that our powers are bound to the body and the body comes in two very different forms, a male and a female. Only when we choose one of them can we access the true powers of the body. There is another and deeper reason why we cannot blossom with our inborn talents if we deny our gender and sexuality: the life energy itself seems to be polarized. The most fundamental energy of life, our basic driving force, has a polar nature. So the appearance of the two sexes is mirroring a very fundamental feature in life, and only when you open yourself for this dimension can you experience true joy.

To accept our own sex, we need our body and sex to be accepted by our mother and father. The need affiliated with the gender-axis is *acceptance*. I can support you on this axis if I give you my *acceptance of your body and gender*. I give this acceptance as a touch done with the intention of acceptance. I can help myself in this dimension by accepting myself.

## DISCUSSION

Love, light (consciousness), and joy are what it takes to express your self fully, and everybody has a huge talent to be expressed and therefore a unique project of self-actualization[3,4,5,6,7,8,9,10,11,12,13,14,15,16,17]. This is the essence of the theory of talent. Love arises from our purpose of life, our life mission[1]. Consciousness brings about the powers of mind, feeling, and body. Joy, the most tricky and philosophically difficult aspect of life, comes from accepting our self completely, including our body, its organs, and its urges, sexuality being the most difficult aspect to accept for many western people.

As the theory of talent is designed for therapeutic use[18,19], what most urgently needs attention with our patients is taken into focus. In a culture where sexuality is fully integrated, the focus might be more on the powers or on the purpose of life and the model might need another presentation. In this model, accepting our gender and sexuality becomes as important as knowing our purpose of life and having the powers of mind, feelings, and body.

The concept of talent is, like the concept of the ego and the concept of the true self, as old as man himself. Many ancient scriptures, the Vedas presumably being the first known source, contain detailed analyses of the existential dimensions of man, their structures, layers, and complicated internal relationships.

The problem with all these complex and often highly composite models is that they are not very easy to use in the modern, holistic medical clinic, where efficient therapy and fast results are a must[20,21,22,23].

Our aim has been to develop the most appropriate models for obtaining these clinical results. What we need for clinical use seems to be very simple, easily communicable, highly useful models and theories and not the very smart and complicated models. We actually need models so simple that they can be explained to every patient in about five minutes together with the existential core concepts[16]. It is our experience that the simple models presented in this series of papers can guide the holistic, existential therapy successfully[20].

The reason to prefer simple models as opposed to complicated ones is also philosophical. The famous principle of Occam's rage tells us that the simplest model or theory that does the job is the most valuable and most true. Another argument for simplicity is the now classical, aesthetic argument of beauty in science. Yet another is the mnemotecnical argument: A simple model is to be remembered and taken into use, a complex model will be forgotten in ten minutes. Of all these arguments, the practical argument seems to be by far the most convincing for a physician or therapist who is anxious to help his patients.

We have tested this with around 200 patients, and have found that it actually makes the concepts and the fundamental existential dimensions much more easy to understand for most patients than the previous models[8,9,24,25]. We know that the model suggested in this paper is so simple that it might be taken as an insult to the much more brilliant and complex models of the ego and the self, especially the famous one by Sigmund Freud and Carl Gustav Jung[8]. As we are pleased with the clinical results from using our new, simpler models[25], we hope that the value of them will be put to a test by our medical colleagues, familiar with the brilliant, but more complex, acknowledged models and theories of human existence.

It is important to note that the barriers that limit one's realization of his or her talents always seems to be in one or more of the existential dimensions of the theory of talent: love or knowing one's self; power, or mastering mind, feelings, and body; and gender, or accepting one's self fully including the body, its organs, and sexuality. When it comes to our patients, the most normal is to have problems in all three areas. If you do not relate to your inner life and existence at all, but only see the external world, you will never discover your own talents to begin with. Not knowing your own talents, you will never be able to grow as a person and in your love for life and others.

## A CASE STORY AND WORKING WITH THE GENDER-AXIS

The theory of talent is useful in many different situations where the physician suspects the origins of the symptoms as psychosocial factors. The theory of talent allows for a fast checking on the existential dimensions to see where the problem presumably is. Using the techniques of holding and processing[18,19], facilitating the holistic process of healing, the problems are often easily solved as the following case demonstrates.

> *Female, aged 33 years with vaginism at her seventh session. The patient relates that immediately when intercourse begins she experiences pain. When she was 17 years old, she tried "a thousand times" to have intercourse with her boyfriend, but was unable to do so. The physician who examined her diagnosed vaginism, which she still suffers from, although today she is able to have intercourse.*
>
> *EXERCISE: Do not accept him until you really have desire. Caress in all other ways first. On the couch, we work on serious chronic tensions in the part of the adductor brevis muscle [one of the femur adductors], which inserts on the pubic bone. Along the way, she relates that when she was 14 years old, she would lie in bed masturbating for two and a half hours at a time and she was sure that she was the only one from school who did it. Her very strong desire was then suppressed so that she did not even feel desire during petting, until her boyfriend forced her to go and see the physician when she was 17. We talk about such strong enjoyment being a great talent, which must be administered consciously. It is a great gift, but induces great resentment if it is not controlled (=*

*condemnation as cheap, a tart, etc.). We work on her shame, guilt, and self-condemnation, which was very marked, and the muscles are slowly loosened.*

*At the eighth session with the sexological procedure of acceptance through touch, it is going really well for her — everyone notices that she is well. Had a period last Saturday — regularly now for the third time in a row 29/5 with normal amount of menstrual bleeding instead of blood "pouring out". Was dumped just after the last session by her boyfriend, which was not much fun. We let go of the boyfriend "with the roll" [this little letting-go technique comes from biodynamics and is really good: Hold onto a kitchen roll, think about what you want to let go, and then let go of the kitchen roll infinitely slowly]. On the couch we work on acceptance of her sex — her hand right down against the vulva, mine supportively on top. We talk about it perhaps being her purpose in life to bring joy and happiness — and that makes her completely desperate and unhappy. If she could choose a talent, it would be to be a leader of large gatherings. "It's so unfair that I did not become a man," she says. She relates that her mother and father thought she was a boy and she was to have been called Brian. There is terribly great charge in this, which is released.*

Please note the relation between her solving the problem and discovering one of her true talents here: talking to other people. What we see here is a great effect of an extremely simple technique: giving acceptance through touch. The sudden, completely spontaneous recognition that she was to have been a boy, with the serious consequences this has had for her in the form of self-condemnation and suppression of her own sex, is surprisingly common. The techniques derived from the theory of talent are often simple, direct, and highly effective. In this case, the focus on her acceptance of her physical sex brings her to the insight within a few hours of holistic therapy. Because of the sexual taboo, she has apparently never received this acceptance previously. We often see, as is also the case here, that menstrual periods become far more regular and there is far less bleeding, when the woman has her relationship with her genitals and her sexuality normalized. Menstrual pain also disappears in many cases.

## CONCLUSION

Everybody has a unique talent. To express our full potential, we most know this essence of our self intimately. We must be genuine in our acceptance of all aspects of life, including sex. We must use all the powers of our life to bring our talent into use in our world, in an effortless and joyful way. Effort is not needed when the power is sufficient and the intent is clear. To be able to thrive and function in life we must know and acknowledge our true self and true human nature. A sound person is good hearted, energetic, and beautiful. A happy life is well intended, conscious, and joyful. It flows[26].

In our natural state of being, we are talented beyond words. A human being who knows and masters his or her core talent is truly a gift to the world. The nurse or physician who encourages the patient to realize him- or herself in an acknowledging, aware, respectful, caring, and accepting way, is not only helping the patient, she or he is helping the world.

Simple models and theories of existence, like the theory of the ego and the theory of talent, seem to be a great help in the holistic clinic, facilitating the holistic process of healing by bringing the patient back to his natural, happy, healthy, and talented state of being.

## BIOSKETCHES

**Søren Ventegodt, MD,** is the Director of the Quality of Life Research Center in Copenhagen, Denmark. He is also responsible for a Research Clinic for Holistic Medicine in Copenhagen and is a popular speaker throughout Scandinavia. He has published numerous scientific or popular articles and a number of books on holistic medicine, quality of life, and quality of working life. His most important scientific contributions are the comprehensive SEQOL questionnaire, the very short QoL5 questionnaire, the integrated QOL theory, the holistic process theory, the life mission theory, and the Danish Quality of Life Research Survey, 1991–94 in cooperation with the University Hospital of Copenhagen and the late pediatric professor Bengt Zachau-Christiansen. E-mail: ventegodt@livskvalitet.org. Website: www.livskvalitet.org/

**Niels Jørgen Andersen, MSc,** Professor, Department of Innovation and Economic Organization, Norwegian School of Management. This department conducts research and provides teaching in central topics related to innovation, business development, management of global companies, business history, and economic organization. Research activities within the Department are related to four core subjects within the discipline: business history, cooperative organizations, business development and entrepreneurship, and finally studies of industries with a special focus on the electricity industry. He is also the dynamic chairman of the nonprofit organization Stiftelsen Holistisk Medisin Scandinavia, which aims to support the scientific development, research, and documentation of complementary and holistic medicine in Scandinavia. E-mail: niels.j.andersen@bi.no. Website: www.bi.no/users/fgl93013/

**Joav Merrick, MD, DMSc,** is Professor of Child Health and Human Development affiliated with the Zusman Child Development Center, Division of Pediatrics and Community Health at the Ben Gurion University, Beer-Sheva, Israel; the Medical Director of the Division for Mental Retardation, Ministry of Social Affairs, Jerusalem; and the Founder and Director of the National Institute of Child Health and Human Development. He has numerous publications in the field of child and human development, rehabilitation, intellectual disability, disability, health, welfare, abuse, advocacy and prevention. Dr. Merrick received the Peter Sabroe Child Award for outstanding work on behalf of Danish Children in 1985 and the International LEGO-Prize ("The Children's Nobel Prize") for an extraordinary contribution towards improvement in child welfare and well being in 1987. E-mail: jmerrick@internet-zahav.net. Website: www.nichd-israel.com
